# Drillers and mill operators in an open-pit gold mine are at risk for impaired lung function

**DOI:** 10.1186/s12995-016-0114-9

**Published:** 2016-05-23

**Authors:** Denis Vinnikov

**Affiliations:** Department of Internal Medicine, Occupational Diseases and Hematology, Kyrgyz State Medical Academy, Akhunbaev street 92, Bishkek, 720020 Kyrgyzstan

**Keywords:** Occupational, Mining, Spirometry, Screening, Workplace

## Abstract

**Background:**

Occupational studies of associations of exposures with impaired lung function in mining settings are built on exposure assessment and far less often on workplace approach, so the aim of this study was to identify vulnerable occupational groups for early lung function reduction in a cohort of healthy young miners.

**Methods:**

Data from annual screening lung function tests in gold mining company in Kyrgyzstan were linked to occupations. We compared per cent predicted forced expiratory volume in one second (FEV_1_), forced vital capacity (FVC) and FEV_1_/FVC between occupational groups and tested selected occupations in multivariate regression adjusted for smoking and work duration for the following outcomes: FEV_1_ < 80 %, FEV_1_/FVC < 70 % and both.

**Results:**

1550 tests of permanent workers of 41 occupations (mean age 40.5 ± 9.2 years, 29.8 % never smokers) were included in the analysis. The mean overall VC was 103.0 ± 12.9 %; FVC 109.1 ± 13.0 % and FEV_1_ 100.2 ± 25.9 %. Drillers and smoking food handlers had the lowest FEV_1_%. In non-smokers, the lowest FEV_1_ was in drillers (94.9 ± 11.3 % compared to 115.2 ± 17.7 % in engineers). Drillers (adjusted odds ratio (OR) 1.53 (95 % confidence interval (CI) 1.11-2.09)) and mill operators (OR 2.01 (1.13-3.57)) were at greater risk of obstructive ventilation pattern (FEV_1_/FVC < 70 %).

**Conclusions:**

Drilling and mill operations are the highest risk jobs in an open-pit mine for reduced lung function. Occupational medical clinic at site should follow-up workers in these occupations with depth and strongly recommend smoking cessation.

## Background

Occupational exposures are linked to a number of respiratory conditions, such as chronic obstructive pulmonary disease (COPD), and population studies identified those exposures to account for 10-15 % of the burden of COPD [1–3]. In workplaces, exposure to dust, vapors and gases comes into play with smoking, and significant number of smokers in dusty workplaces will eventually develop COPD [4]. Therefore, in hazardous enterprise, primary prevention is directed to minimizing exposure to dust and proper use of personal protective equipment.

Studies of occupational role of dust were mainly based on either self-reported or measured exposure assessment, and in those analyses, epidemiological studies with proper industrial hygiene data and exposure assessment would have the biggest weight [3]. The alternative to such exposure measurement approach [5] could be particular workplace assessment, where exposures come into complex interaction, and this makes multifactorial cause of occupational COPD plausible. For practical reasons, occupational intervention to detect and combat early lung function impairment may be based on workplace assessment with or without exposure data. Such approach has identified occupations with high risk, and those may be machine operators, construction trades and other related types of work [6]. In mining, which is by itself usually a high-risk enterprise (mining at altitude), knowing which occupations and workplace entail the greatest hazard to a worker’s respiratory health is important. Identifying vulnerable groups in a mining setting can then guide proactive monitoring.

Annual screening, where spirometry is mandated, can be one of the tools to detect lung function impairment at very early stages to guide worker placement [2]. Spirometry has been shown to identify early lung function changes in those exposed to mineral dust [7], however identifying vulnerable groups at mining site in the absence of industrial hygiene data is still challenging. Just knowing that dust is associated with COPD is not sufficient for selecting high-risk workers for more thorough monitoring. Thus, the aim of this study was to identify vulnerable groups for early lung function reduction in a cohort of healthy young miners employed for gold mining operation, where industrial hygiene data do not exist.

## Methods

### Study design and population

This analysis used lung function data of workers at a high-altitude gold mine operating at an altitude of 3800–4500 meters above sea level and situated in the Tyan Shan mountain range in Kyrgyzstan. This was an open pit mine, and gold was extracted on site. Local employees worked two- or three-week 12-h per day shifts at altitude and commuted to their homes for two or three more weeks at low or middle altitude. All high-altitude workers underwent pre-employment and annual screening carried out in a specially designated clinic in Bishkek, including lung function testing. Data collected at this examination included smoking habits, life style attributes, former and current medical history. Such annual screening also comprised physical examination by eight narrow specialists, supplemented by ECG, frontal chest X-ray, blood work, urine test, lipid, liver enzymes and nitrogen metabolism biochemical tests, audiometry, night vision test and other tests upon indications (e.g.*,* cardiac ultrasonography). This study was approved by the Committee on Bioethics of Kyrgyz State Medical Academy.

Dataset analyzed in this study were all spirometric tests done during one calendar year, when ideally all workers should be enrolled, and 2102 tests in total were performed. In general, people working at the mine were healthy and fit, as there was a list of legally mandated contraindications for employment at high altitude covered by the Regulation 225 in 2011 (formerly Order 70 of 2004). Only local subjects working at the mine site and a large marshalling yard located 200 km away from the mine were included (foreign nationals excluded), belonging either to Kyrgyz (93.6 %) or other ethnic groups (6.4 %). Job lists with relevant departments for each subject were obtained from human resources department of the company and were not self-reported, accompanying referral letter for annual screening.

### Occupations

The list of employed occupations was mainly dictated by the specific attributes of open-pit mining. Drivers were those operating heavy-duty vehicles, such as mine trucks, graders, shovel machines, loaders, and bulldozers. Mechanics and other maintenance staff were employed in the on-site workshops, repairing machines and assembling new vehicles from parts. Food handlers were involved in various stages of storing, cooking and distributing food to workers at site. Drillers were principal operators and their assistants who control drilling equipment right in the pit. Cleaners worked mainly in the camp doing all types of daily cleaning in dwelling premises and non-residential areas in the camp. Samplers and surveyors were specific mining occupations working in an open pit. Lab technicians were those operating chemical analytical lab inside the mill. Finally, mill operators was a heterogeneous group of workers involved in various stages of gold extraction process within the mill.

### Spirometry

Spirometry was performed in the occupational clinic located in Bishkek, always in the midpoint of a two-week off-duty period and typically in the morning. Lung function test was done with MicroMedical MicroLab (UK) equipment. The subject was in a standing position, and asked to refrain from smoking at least for two hours prior to the test, as prompted by spirometry guidelines in Kyrgyzstan [8]. All tests were performed by the same staff, who were regularly trained, equipment was daily calibrated and biological quality control was performed once a month. At least three vital capacity maneuvers and at least three forced vital capacity maneuvers with reproducibility less than 4 % were required. Because no reference spirometry data existed for Kyrgyz population, we used the European Coal and Steel Community (ECSC) reference equations values to calculate percent predicted values for forced expiratory volume in one second (FEV_1_) and forced vital capacity (FVC) [9]. We also measured vital capacity (VC), peak expiratory flow (PEF), and flows at 75 %, 50 % and 25 % of the remaining FVC (MEFs). Quality control was performed by either one of two physicians trained in such testing. The best curves were those with maximal (FEV_1_ + FVC).

### Smoking status verification

Workers were defined “never-smokers” if they answered “No” to the question “Have you ever smoked a cigarette?”. Should they answer “Yes”, but stopped smoking at some point before, they were “Former smokers”. Those smoking daily at a time when study was carried out were defined as “Current smokers”. Self-reported current smoking status was verified by exhaled carbon monoxide (CO) measurement performed just prior to spirometry. A Smokerlyzer CO (Bedfont, UK) was used for this testing; readings below 10 ppm were interpreted as confirmatory of non-active smoking status. Those having CO level 10 ppm and above from any self-report category were treated as “Current smokers”.

### Statistical analysis

Exposure metrics were jobs obtained through the list from the HR-department, which were coded into 41 occupations. In a univariate analysis all codes were analyzed separately, however in regression models we grouped relevant occupations in bigger groups, such as all drivers in one category. Outcome measures were selected lung function indices, such as VC, FVC, FEV_1_, FEV_1_/FVC, PEF and MEF_25–75_. For this analysis, we calculated per cent predicted values of lung function indices, which were based on age, height, and sex, also corrected for ethnicity. For univariate comparisons, we used Wilcoxon test to determine whether differences in lung function indices between occupations and between smokers and non-smokers were due to chance only. For regression models we created three outcomes measures, which we tested separately in multivariate models: 1) reduction of FEV_1_ to less than 80 % (reference – 80 % and more); 2) reduction to FEV_1_/FVC to less than 70 % (reference – 70 % or more); and 3) reduction of FEV_1_ to less than 80 % and reduction to FEV_1_/FVC to less than 70 %. We adjusted our regression models for smoking and work duration, as those were identified as potential confounders on a pathway from an exposure to an outcome. Because %predicted values already account for age and sex, their effect was eliminated and the models were not adjusted for those variables. Smoking variable was coded as never-smokers vs. ever-smokers (reference). We also performed regression analysis in never smokers only, but due to loss of power, we only report them briefly. We used NCSS 9 (Utah, USA) software for all tests. The effect measure in regression models of this cross-sectional study was odds ratio (OR) with relevant 95 % confidence interval (CI).

## Results

A total of 2102 spirometry tests were performed during the period of 2010. Of those, 344 were new hires, having spirometry tests at their pre-employment screening. Because their previous employment record and exposure history were not available, they were excluded from the analysis. Of remaining 1758 employees, spirometry test records of 208 employees were incomplete, yielding 1550 tests available for final analysis. The mean age of this predominantly male workforce (87.5 %) was 40.5 ± 9.2 years (Table 1).

**Table 1:** Study participants’ profile

Variable	
N	1550
Age, years	40.5 ± 9.2
Male/female, N	1357/193
BMI, kg/m^2^	25.8 ± 3.7
Working at high-altitude site, N (%)	1477 (95.3 %)
*Smoking*	
Current smokers, N (%)	627 (40.5 %)
Cigarettes a day	9.2 ± 4.8
Duration of smoking, years	13.7 ± 7.8
Ex-smokers, N (%)	460 (29.7 %)
Never smokers, N (%)	463 (29.8 %)
*Spirometry*	
VC, % pred.	103.0 ± 12.9
FVC, % pred.	109.1 ± 13.0
FEV_1_, % pred.	100.2 ± 25.9
FEV_1_/FVC, %	76.5 ± 20.3
PEF, % pred.	107.5 ± 16.7
MEF_50_, % pred.	77.7 ± 24.1
*Occupations*	
Mechanics, N (%)	242 (15.6)
Mine truck drivers, N (%)	221 (14.3)
Bulldozer, Grader and Loader/shovel operators, N (%)	128 (8.3)
Cleaners, N (%)	99 (6.4)
Security staff, N (%)	96 (6.2)
Drillers, N (%)	88 (5.7)
Office staff, N (%)	82 (5.3)
Other drivers, N (%)	72 (4.6)
Engineers, N (%)	61 (3.9)
Food handlers (kitchen), N (%)	58 (3.7)
Mill operators, N (%)	48 (3.1)
Warehouse staff, N (%)	45 (2.9)
Blasters, N (%)	41 (2.6)
Lab technicians, N (%)	36 (2.3)
Welders, N (%)	34 (2.2)
Samplers, N (%)	23 (1.5)
Geologists, N (%)	18 (1.2)
Grinder operators, N (%)	14 (0.9)
Surveyors, N (%)	11 (0.7)
Other, N (%)	133 (8.6)

‘Other drivers’ include powertrucks, passenger trucks and conveyance vehicles; ‘Office staff’ include trainers, accountants, management, administrators and interpreters. Data presented as mean ± standard deviation

More than 90 % of personnel worked at high-altitude site, whereas the rest were employed at a marshalling yard at middle altitude, and the workforce of this yard was mainly made of powertruck drivers, security personnel and warehouse staff. The occupational profile of the main operation site was more diverse. Drivers and vehicle operators were the most prevalent occupations, and when combined with maintenance (mechanics, electricians and related occupations), altogether they made 45 % of staff. Smoking prevalence was high, and never smokers made only 29.8 % of the cohort. Smoking intensity was not high, though, and an average smoker smoked 9.2 cigarettes a day.

In general, this cohort were mainly healthy men with excellent lung function. Both volume and flow parameters were above 100 % predicted, and men did not differ from women in spirometric %predicted values. In total, this cohort comprised workers of 41 occupations, which we grouped into larger groups for further lung function analyses. We selected groups with the cumulative prevalence of three or more percent and their selected spirometry data are presented in Table 2. Cleaners showed significantly greater FEV_1_% when compared to any other occupation, and the difference with the poorest lung function group (drillers) reached 7.5 % (*p* < 0.001). Similarly, drillers had still the worst lung function, when only non-smokers of each occupational group were included in the analysis. Finally, FEV_1_% difference in non-smoking drillers increased to 10.8 % when compared to non-smoking cleaners (*p* < 0.001).

**Table 2:** Spirometry data of employees with selected occupations (prevalence 3 % or more)

Occupations	FVC, % pred.			FEV_1_, % pred.			FEV_1_/FVC, %		
	Overall	NS	S	Overall	NS	S	Overall	NS	S
Heavy-duty vehicles operators	107.8 ± 13.3	106.7 ± 13.8	108.1 ± 13.1	98.0 ± 12.9	98.1 ± 13.5	97.9 ± 12.8	75.1 ± 6.5	76.0 ± 5.4	74.9 ± 6.7
Other drivers	109.1 ± 13.6	109.4 ± 14.8	108.9 ± 13.3	100.3 ± 13.2	101.3 ± 13.3	99.9 ± 13.2	75.2 ± 6.0	75.9 ± 5.3	75.0 ± 6.3
Mechanics	109.1 ± 12.5	110.6 ± 11.2	108.7 ± 12.8	99.3 ± 12.4	101.3 ± 12.1	98.8 ± 12.5	75.9 ± 7.0	77.7 ± 7.0	75.9 ± 6.9
Cleaners	116.8 ± 13.4	116.9 ± 13.5	116.6 ± 13.4	105.3 ± 12.4	105.7 ± 12.6	103.6 ± 11.7	77.5 ± 5.7	77.9 ± 5.8	75.8 ± 5.1
Security staff	109.8 ± 11.2	110.0 ± 10.2	109.7 ± 11.6	99.7 ± 11.2	101.1 ± 11.0	99.1 ± 11.2	75.2 ± 5.0	76.4 ± 6.3	74.7 ± 4.4
Drillers	105.7 ± 12.5	103.5 ± 11.9	106.5 ± 12.6	97.8 ± 13.2	94.9 ± 11.3	98.8 ± 13.7	76.7 ± 7.2	75.8 ± 4.7	77.0 ± 7.9
Office staff	108.5 ± 13.3	108.0 ± 13.5	108.8 ± 13.3	100.4 ± 12.9	101.8 ± 13.9	99.6 ± 12.3	77.7 ± 7.1	79.1 ± 8.5	76.3 ± 5.9
Engineers	108.2 ± 13.2	110.8 ± 17.4	105.3 ± 11.8^a^	101.2 ± 16.0	115.2 ± 17.7	96.6 ± 12.8^a^	77.7 ± 7.2	80.8 ± 3.6	76.7 ± 7.8
Food handlers	113.0 ± 13.1	115.4 ± 12.6	106.6 ± 12.8^a^	99.4 ± 11.8	101.6 ± 12.3	93.9 ± 8.2^a^	75.5 ± 7.1	75.8 ± 7.4	74.8 ± 6.4
Mill operators, including grinder operators and metallurgists	108.5 ± 12.4	109.9 ± 12.1	107.8 ± 12.8	98.7 ± 13.4	100.5 ± 14.7	97.7 ± 12.9	75.0 ± 7.4	75.1 ± 7.0	74.7 ± 7.6

*NS* never smokers, *S* smokers and ex-smokers. Heavy-duty vehicles operators include mine truck operators, bulldozer, grader and loader/shovel operators; ^a^ significant difference when compared to never-smokers. Data presented as mean ± standard deviation

Of note, we could not detect significant differences when comparing never-smokers with smokers within any occupational group (Table 2), except engineers and food handlers. Only in these two occupational groups ever-smokers had significantly lower FVC% and FEV_1_% compared to never-smokers, and non-smoking engineers showed the highest FVC% and FEV_1_%: their FEV_1_% was as high as 115 %, and the absolute difference of non-smoking engineers with non-smoking drillers exceeded 20 %.

We wished to further investigate the interplay of occupation with smoking in the association with poor lung function using regression model. When the two confounders were included in the model, most occupations were not significantly associated with selected outcomes, except drilling and mill and grinder operation. Table 3 shows that drillers had statistically significant 53 % higher risk of obstruction (FEV_1_/FVC < 70 %), whereas work at the mill and grinding increased the risk two-fold (also for FEV_1_/FVC < 70 %). This table only shows models with adjusted regressions. We further selected groups with marginally high or close to significantly high risk to test using cleaners as reference group (as the lowest risk occupation). Because of loss of power, most of these models became unstable, however when drillers were compared to cleaners, the OR of FEV_1_ < 80 % was 10.2 (95 % CI 1.05-97.80). Similarly, in mill workers the OR of FEV_1_/FVC < 70 % was 3.81 (95 % CI 1.37-10.44). Finally, to demonstrate isolated effect of work duration on obstruction, we tested it as predictor for three metrics of obstruction as in Table 3. In all cases the effect of work duration was very small and even non-significant in two metrics of three (ORs 1.06 (95 % CI 1.02-1.10); 1.06 (95 % CI 0.94-1.19); and 1.03 (95 % CI 0.90-1.19).

**Table 3:** Regression models of an association between an occupation and selected spirometric outcomes

Exposures (occupations)	FEV_1_ < 80 %	FEV_1_/FVC < 70 %	FEV_1_/FVC < 70 % and FEV_1_ < 80 %
Drillers	1.46 (0.69–3.11)	1.53 (1.11–2.09)	1.53 (0.60–3.92)
Drivers	1.24 (0.80–1.91)	1.18 (0.88–1.58)	1.03 (0.58–1.84)
Mechanics	0.63 (0.33–1.19)	0.95 (0.65–1.37)	0.80 (0.37–1.71)
Cleaners	0.14 (0.02–1.05)	0.51 (0.24–1.09)	0.26 (0.03–1.93)
Office staff and engineers	1.30 (0.61–2.75)	0.90 (0.51–1.59)	1.68 (0.71–4.02)
Food handlers	1.18 (0.41–3.40)	1.11 (0.53–2.33)	0.99 (0.23–4.25)
Mill operators including grinders	0.73 (0.22–2.36)	2.01 (1.13–3.57)	1.29 (0.39–4.24)
Security staff	0.60 (0.22–1.68)	0.84 (0.47–1.51)	0.28 (0.03–1.80)

data are presented as adjusted for smoking and work duration odds ratios (OR) with relevant 95 % confidence intervals (CI); group ‘Drivers’ includes all relevant operators. Reference groups in each model are all other occupations combined

## Discussion

This was a cross-sectional study of lung function at mandatory annual screening of open-pit gold mine in Kyrgyzstan with the aim to ascertain most vulnerable working groups for early lung function impairment in healthy young workers. In general, workers in various workplaces within the company had excellent spirometry, with all main parameters exceeding 100 % predicted values. Even with fairly high overall smoking prevalence, never smoking workers had similar flows with ever-smokers in almost all occupations, which was due to healthy worker effect, when initially most fit subjects were selected for employment. Using regression analysis, we found drilling and work in the mill were significantly associated with FEV_1_/FVC reduction, and working in the mill doubled the risk of obstruction.

Mining is an established setting for occupational respiratory morbidity, and silica exposure in mining venues is associated with silicosis and lung cancer [10, 11]. Mining workplaces also have high dust levels, and exposure to dust in workplace can result in an occupational COPD [12]. Because COPD is a chronic progressive inflammatory condition, the disease develops gradually, and occupational spirometric screening should detect abnormality at earlier stage [13, 14]. When no dust and other exposure measurements are available, screening of high-risk groups may only be feasible based on occupation-specific data to identify most vulnerable workers for more effective follow-up. Since the population attributable fraction of occupational exposures for COPD is around 15 %, early prevention is crucial and should be a cornerstone of an occupational screening clinic routine. Identification of high-risk workplaces could help prevent large number of COPD cases, mostly in never-smokers. A study like this a helpful tool to monitor workers employed in drilling and mill operations as well as to identify workplaces where high-quality dust exposure measurements are needed.

The findings of this study also emphasize the need for better enforcement of workplace control in drilling and in the mill, where exposure to dust, gases and vapors during chemical extraction of gold may be high. Regular dust monitoring program with active dust level reduction are essential to sustain good workforce health in these workplaces. When no full dust elimination is possible, only subjects with excellent baseline lung function should be hired for these positions; however, current legislation in Kyrgyzstan does not prohibit employment of workers with COPD, except workers with “chronic bronchopulmonary diseases” applying for jobs with documented high exposure to silica dioxide. Existing screening regulation does not list specific workplaces, therefore occupational screening program is challenging for an occupational doctor in mining companies. More intense lung function monitoring with relevant clinical assessment could serve an optional way to slow down lung function decline in drillers and mill operators. One of the ways to do so may be lung function test done twice a year in these groups, supplemented with smoking status verification and documentation, followed by strong cessation advice.

This study has a number of limitations. Such occupations of interest as welding, which are known to have high levels of exposure, were not included in the models because of their small sample size. There were very few surveyors, whose field work in an open pit can also predispose them to higher exposure to dust. Another limitation was a significant shift towards healthy workers with excellent lung function and relatively small number of employees with either initial or advanced stage of respiratory conditions. Such selection is a result of healthy worker effect, a typical selection bias in occupational studies. Nevertheless, most pronounced limitation of our epidemiological study was nonexistence of exposure assessment data to relate them to actual workplace data. Historically, exposure data were inaccessible in this mining enterprise, and their introduction in future would dramatically advance risk mapping and guide occupational doctor at site to enhance prevention activities in vulnerable groups. Directed by preliminary findings from this setting, exposure assessment in drilling workplaces and all over the mill should be given priority and are a matter of research in future.

Good occupational practice assumes maximum elimination of exposure and regular surveillance with the aim to improve knowledge, attitudes and behavior of all workers at risk of developing occupational COPD [2]. Primary occupational doctors at site should further enforce annual lung function decline monitoring programs, because accelerated decline more than 10–15 % from baseline should not stay unattended. High altitude by itself poses stress on lung function in those newly employed with accelerated decline [15], and occupational exposures together with smoking will accelerate this decline further. Based on these observations, the highest risk is attributed to newly employed young smoking men in drilling and the mill. They should not only be closely monitored, but strongly advised to cease smoking with effective pharmacological aid and be thoroughly instructed on proper use of personal protection.

This study is noted for a number of strengths. Exposure ascertainment was quite strong, as data on work history and positions were obtained from HR department. Smoking was also accurately measured, because self-reported exposure was verified using biological markers, which is rare for occupational epidemiologic research. Large cohort size in this study, especially of selected occupations, such as heavy-duty drivers, should also be considered a strength, and resulted in a greater power. And finally, using a workplace approach rather than exposure approach enabled to incorporate all the mix of various exposures in a single workplace. Workplaces are very seldom a single-exposure matrix, so just knowing particular exposure measure, such as particulate matter with aerodynamic diameter 10 μm and less (PM_10_) is far not sufficient for full assessment.

## Conclusions

In conclusion, this cross-sectional study of a large sample of workers in an open-pit gold mine has demonstrated that on overall lung function of mine workers was within normal range, and had clear correlations with the workplace. We have identified two workplaces with the highest risk for FEV_1_/FVC reduction, including drillers (operators of drilling machines) and employees in the mill. This should guide regular spirometric surveillance of workers in these occupations and identify subjects at risk at earlier stages of lung function decline.
